# Single‐Cell Transcriptomic Reveals the Involvement of Cell–Cell Junctions in the Early Development of Hypertrophic Cardiomyopathy

**DOI:** 10.1111/jcmm.70366

**Published:** 2025-02-03

**Authors:** Dingchen Wang, Miao Lin, Ruobing Wang, Xiaoran Huang, Yaowen Liang, Xiran Wang, Yuge Chen, Yunfei Gao, Huiming Guo, Huiying Liang, Xin Li

**Affiliations:** ^1^ School of Medicine South China University of Technology Guangzhou Guangdong Province China; ^2^ Department of Emergency Medicine Guangdong Provincial People's Hospital (Guangdong Academy of Medical Sciences), Southern Medical University Guangzhou Guangdong Province China; ^3^ Guangdong Cardiovascular Institute Guangdong Provincial People's Hospital, Guangdong Academy of Medical Sciences Guangzhou Guangdong Province China; ^4^ Medical Big Data Center Guangdong Provincial People's Hospital (Guangdong Academy of Medical Sciences), Southern Medical University Guangzhou Guangdong Province China; ^5^ Guangdong Provincial Key Laboratory of Artificial Intelligence in Medical Image Analysis and Application Guangzhou Guangdong Province China; ^6^ Shantou University Medical College Shantou Guangdong Province China; ^7^ Department of Obstetrics and Gynecology The First Affiliated Hospital of Anhui Medical University Hefei China; ^8^ Zhuhai Precision Medical Center Zhuhai People's Hospital (Zhuhai Hospital Affiliated with Jinan University), Jinan University Zhuhai Guangdong Province China; ^9^ The Biomedical Translational Research Institute Jinan University Faculty of Medical Science, Jinan University Guangzhou Guangdong Province China

**Keywords:** cell–cell junction, endothelial cells, hypertrophic cardiomyopathy, snRNA‐seq

## Abstract

The relationship between the changes in endothelial cell–cell junctions and microvascular abnormalities in the progression of hypertrophic cardiomyopathy (HCM), as well as their potential as early biomarkers, remains unclear. Here, we analysed single‐nucleus RNA‐sequencing data from the left ventricles of 44 health donors and HCM patients. First, we observed that endothelial cell–cell junctions were significantly altered in HCM vascular endothelial cells (ECs), including tight junctions, gap junctions and adherens junctions, especially in capillary ECs. The proposed pseudo‐timing analysis predicted that endothelial cell–cell junctions abnormalities occurred in the early stages of HCM. Second, we verified that endothelial cell–cell junctions disorders occur at early stages of HCM disease progression in two time‐series single‐nucleus datasets of mice. The expression of eight cell–cell junction genes showed an initial increase in the early stage, followed by a slight decrease in the middle stage, and a sharp increase in the later stage. Subsequently, cell communication and transcription factor analysis were used to explore the underlying mechanisms. Furthermore, an early HCM prediction model was developed and independently validated using three mRNA datasets comprising 204 health individuals and HCM patients for the eight genes panel, the accuracy was 0.81 [0.63–0.98]. Finally, we validated this panel in HCM tissues. This study demonstrated in humans and mice that eight cell–cell junction genes were significantly elevated in the early stages of HCM and may be potential biomarkers for the early diagnosis of HCM.

## 1 Introduction

Hypertrophic cardiomyopathy (HCM), a cardiac disorder characterised by left ventricular hypertrophy, is now widely recognised as a more common condition, affecting approximately 1 in every 200–500 individuals [1]. HCM is known to be a significant cause of arrhythmic sudden death, heart failure and atrial fibrillation [2, 3, 4]. Pathological manifestations in the hearts of HCM patients include impaired cardiomyocyte function, enhanced fibroblast activation, chronic inflammation and cell death [5]. While our understanding of genetic factors, clinical course and management of the broad spectrum of HCM has evolved measurably in the past 15 years [6, 7, 8], the molecular mechanisms underlying HCM and the microscale changes during disease progression are not yet fully understood. Current research on HCM mainly focuses on cardiomyocytes and fibroblasts [9, 10], while the crucial role of endothelial cells (ECs) in disease development and changes is often overlooked.

ECs, situated between the blood and the blood vessel wall, directly interact with the bloodstream, serving as guardians of cardiovascular health. ECs play a pivotal role in maintaining cardiovascular homeostasis by regulating blood flow, angiogenesis, monocyte adhesion, and platelet aggregation [11]. Vascular ECs actively participate in cellular barrier processes, establishing continuous complexes of tight and adherens junctions (AJs) along EC‐EC contacts, thus providing a tight and size‐selective barrier [12]. Changes in the expression, localization, and architecture of endothelial cell junctions have been observed in various vascular disorders and conditions, including hypertension, atherosclerosis, and thoracic aortic aneurysm [13, 14, 15]. However, it remains to be determined whether cellular junction processes are involved in HCM.

Within ECs, the vascular barrier is primarily formed by tight junctions (TJs), gap junctions (GJs), and AJs. These junctional complexes play a vital role in maintaining endothelial integrity and safeguarding blood vessels against infiltration and inflammation [16]. TJs are highly dynamic structures composed of transmembrane proteins such as claudins, occludin and junctional adhesion molecules [17]. GJs play a crucial role in mediating cell‐to‐cell communication, comprising specialised structures composed of connexins (Cx) [18]. AJs are ubiquitously distributed along the vascular tree and are expressed in both blood and lymphatic vessels [19]. These structures are formed by transmembrane adhesion proteins of the cadherin family, which mediate homophilic adhesion and can organise into multimeric complexes at cell borders. This study aims to elucidate whether endothelial cellular junction processes are involved in HCM progression and to characterise the alterations that occur during the course of the disease.

Single‐nucleus RNA sequencing (snRNA‐Seq) provides high‐resolution transcriptomic analysis of individual cells and could help explore the association between changes in endothelial cell–cell junctions and microvascular abnormalities in the progression of HCM [20]. In this study, we initially analysed snRNA‐Seq data to classify ECs into eight subgroups and identified aberrant cell junction pathways in inter‐group comparisons. Additionally, we conducted a pseudotime analysis and utilised mouse time‐series data to investigate the predictive role of cell junctions in early disease stages. Finally, we established a disease classification model based on cell junction gene sets, demonstrating its potential effectiveness for early screening. According to our study, targeting endothelial cellular junctions might be a promising strategy for the early screening and monitoring of HCM.

## 2 Materials and Methods

### 2.1 Human Samples

Human samples in this study, myocardial tissues were from healthy donors (control) and patients of HCM. All human samples shared a common age range, The HCM patients within our study were individuals who had undergone surgeries at our hospital due. These samples were meticulously archived in the official tissue biobank at Guangdong Provincial People's Hospital, accompanied by the requisite ethical documentation. The “control donors” refers to individuals who are healthy but lost their lives under various circumstances, such as car accidents. All patients had signed an informed consent form before sample collection.

The study was conducted according to the guidelines of the Declaration of Helsinki, and approved by the Ethics Committee of Guangdong Provincial People's Hospital (No. KY‐Q‐2022‐139). Informed consent was obtained from all subjects involved in the study.

### 2.2 Data Sources

We utilised public datasets to conduct our research. The human snRNA‐seq data on Figureshare (https://doi.org/10.6084/m9.Figureshare.c.5777948.v2) [21]. and the Broad Institute's Single Cell Portal (project ID SCP1303: https://singlecell.broadinstitute.org/single_cell/study/SCP1303) [22]. These datasets included 10 HCM patients and 3 health individuals from Fuwai Hospital, and 15 HCM patients and 16 health individuals from the Myocardial Applied Genetics Network (MAGNet; www.med.upenn.edu/magnet). The MAGNet samples included both non‐failing (NF) and failing heart tissues from patients. In brief, NF heart tissue samples were obtained from organ donors without a history of heart failure. Failing heart samples were collected from explanted hearts of donors undergoing heart transplantation. The demographic information of these samples, including age, gender, and clinical characteristics, has been detailed in previous publications [21, 22]. The transverse aortic constriction (TAC) mouse snRNA‐seq data was sourced from GSE120064 which included representative stages at 0, 2, 5, 8 and 11 weeks after TAC [23]. The data on genetic fate tracing of cardiac ECs, collected at 0, 2 and 4 weeks from 12 CreER; td‐Tomato mice‐lines, can be accessed at GSE166403 [24]. Lastly, three clinical mRNA datasets for the classification model are available on GSE36961 (discovery data), GSE160997 (External Validation 1) [25], and GSE130036 (External Validation 2) [26].

### 2.3 General Clustering

Standard procedures for filtering, variable gene selection, dimensionality reduction, and clustering were carried out using Seurat v4.1.1 [27]. The following quality control steps were performed: (i) genes expressed in fewer than 3 cells were excluded; (ii) cells that expressed fewer than 200 genes (low quality), and cells that expressed over 5000 genes (potential doublets) were excluded from further analysis; (iii) cells with fewer than 10% mitochondrial RNA content, more than 500 unique UMI counts or more than 15,000 unique UMI counts were removed [28].

**FIGURE 1:**
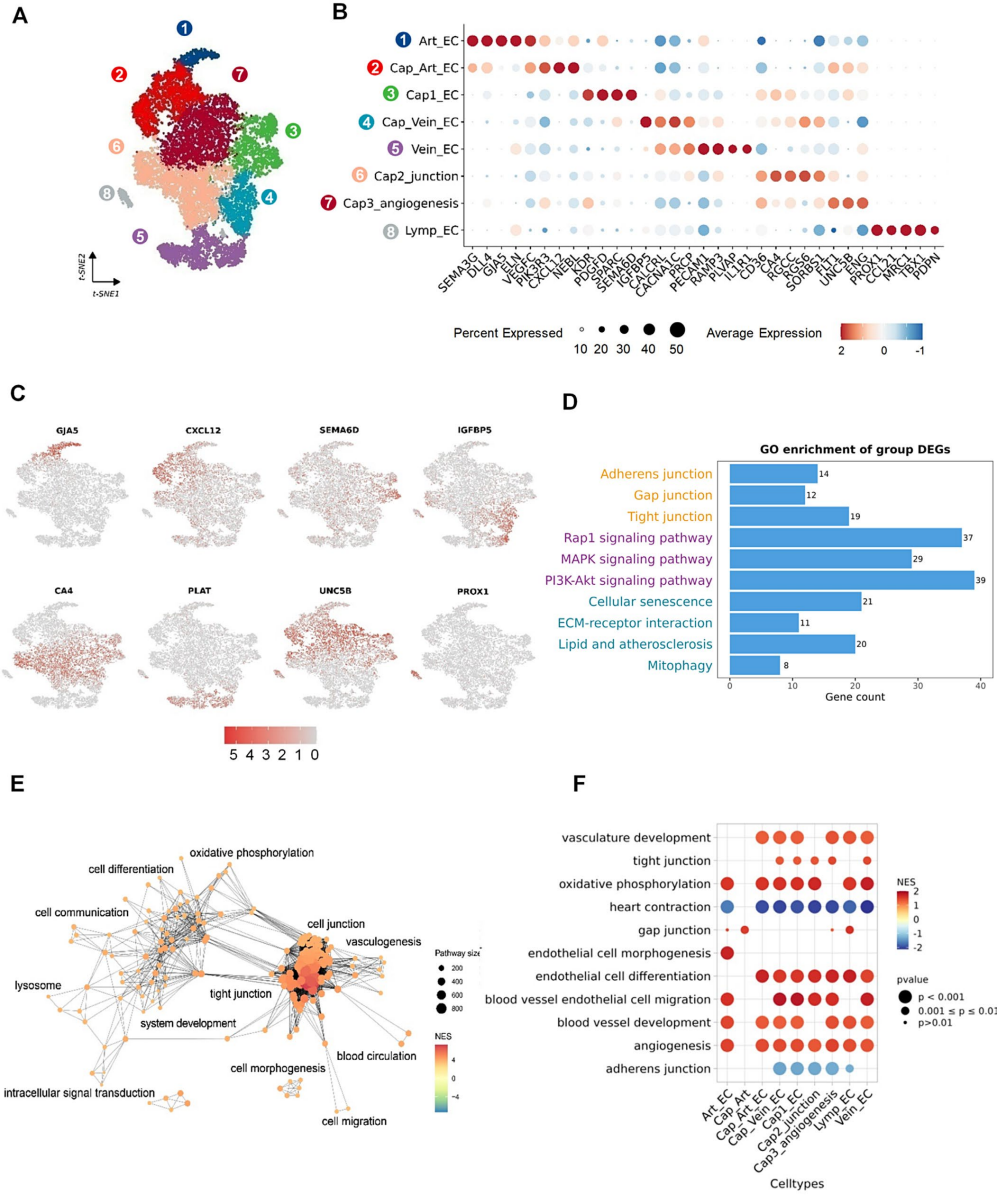
Aberrant cell junction pathways identified in endothelial cells (ECs) of HCM (A) t‐SNE diagram showing the division of ECs into 8 subpopulations. (B) Dotplot presenting marker genes for eight subgroups, colour indicating average expression and size indicating the percentage of cellular expression. (C) Featureplot demonstrating subpopulation distribution, each marker gene represents a subpopulation. (D) GO enrichment analysis of differential genes between health and HCM groups. (E) Pathway enrichment topology plot of HCM and health groups. NES (Normalised Enrichment Score) demonstrated the degree of enrichment of gene sets. The size of the dots represented the size of the gene sets. (F) Enrichment analysis of DEGs in the eight subpopulations using GSEA. The size of the scatter points symbolised the significance level, categorised into three levels: *p* < 0.01, 0.01 < *p* < 0.05 and *p* > 0.05. The colour of the points was filled based on the NES.

Counts were log‐normalised and then scaled by linear regression against the number of reads. Variable genes (features = 2000) were selected using a threshold for dispersion, with *z*‐scores normalised by expression level. To reduce the dimensionality of the dataset, we applied principal component analysis (PCA) on the highly variable genes. This was done using the RunPCA function with default settings on data that had been linearly transformed and scaled using the ScaleData function. Additionally, the percentage of mitochondrial gene expression was regressed out to mitigate its effects. To minimise batch effects across samples and experiments, the canonical correlation analysis (CCA) method in Seurat was utilised. A shared‐nearest‐neighbours graph was constructed based on the metric of the Euclidean distance in the low‐dimensional subspace. Cells were visualised using a 2‐dimensional tSNE on the same distance metric (Res = 0.5, dims = 25). Cell types were assigned to each cluster of cells using the abundance of known marker genes (Figure 1B).

The broad classification of vascular ECs was annotated using commonly recognised endothelial cell markers such as VWF, PTPRB and PECAM1. The subtypes of ECs were identified based on previous studies and widely accepted markers. For example, lymphatic ECs were marked by PROX1 and TBX1, while capillary vein ECs were identified using markers such as GFBP5 and CALCRL. The visualisation of cell clustering was done using the R package jjAnno (v 0.0.3), an annotation package designed for enhancing GGplot plots. Detailed information about the package can be found at GitHub and its manual (https://github.com/junjunlab/jjAnno).

### 2.4 Marker Gene Definition

The FindMarkers (or FindAllMarkers) function was used to identify DEGs by comparing cells from a subcluster to others. The FindAllMarkers function was used with parameters, min.pct = 0.25, thresh.use = 0.25, only.positive = TRUE and return.thresh = 0.05, the Wilcoxon rank‐sum test was employed as the statistical method. To capture genes with subtle differences but potential biological significance, DEGs were filtered based on a minimum log fold change of 0.25. For differential expression analysis between sub‐groups, each sub‐group of cells was compared against all other cells collectively.

### 2.5 Endothelial Cell Subset Analysis

ECs were re‐clustered using methods described above and the following parameters: features = (2000), dims = 30, Res = 0.4. To detect genes expressed specifically in each cluster, the clusters were compared pairwise using the Seurat “FindAllMarkers” function to test genes with > 0.25‐fold difference (log‐scale) on average between the cluster of cells and detectable expression in more than 25% of cells in either of the clusters. Differential genes were identified using a significance cutoff of *q* < 0.05. For DEGs enrichment analysis, biological processes enrichment of significantly upregulated genes in each cluster of ECs were performed using the R package clusterProfiler [29]. Results were visualised using clusterProfiler and ggplot2 R packages.

### 2.6 Trajectory Inference and Pseudotime Calculation

Monocle2 (v2.26.0) [30, 31, 32] and slingshot (v2.12.0) [33] was employed to investigate pseudotime trajectories of cells. DDRTree was utilised to perform the dimensionality reduction necessary for constructing the trajectory. Branches in the cell trajectory were used to represent cells that showed alternative gene expression patterns. Then the cell differentiation trajectory was inferred with the default parameters of Monocle2 after dimension reduction and cell ordering. We used the “differentialGeneTest” function to derive DEGs from each cluster and genes with a *q* < 0.01 to order the cells in pseudotime analysis.

### 2.7 Cell–Cell Interaction Analysis

To analyse cell–cell interactions between different cell types, we used CellChat [34] (https://github.com/sqjin/CellChat; commit ID 9e1e605) R packages to identify significant ligand‐receptor pairs within ECs. For both HCM and health ECs, the cell types' specific receptor‐ligand interactions were identified based on the specific expression of a receptor by one cell type and a ligand by another cell type. The interaction score was calculated as the average expression value of individual ligand‐receptor pairs in the interacting cell types. Subsequently, the netVisual circle function was employed to display the effectiveness of cell–cell communication networks from the intended cell cluster to various cell clusters.

### 2.8 Diagnostic Model for Hypertrophic Cardiomyopathy

We collected three sets of left ventricular mRNA data to build a robust classifier that can identify patients with HCM, one as a discovery set for model construction, and 2 datasets as external validation set to evaluate model performance. We ultimately incorporated eight cell–cell junction genes identified in the research into the model.

Model training then used the discovery cohort alone, split into training/validation/testing using both 9:3 (70/10/20) and 11:1 (70/10/20) splits. We initially based our choice of machine learning classification methods on the 8 and 19 DEGs, comparing the performance metrics of 12 different classification methods. These included CatBoostClassifier, XGBClassifier, AdaBoostClassifier, RandomForestClassifier, GaussianNB, LGBMClassifier, LogisticRegression, GradientBoostingClassifier, MLPClassifier, DecisionTreeClassifier, SVC and KNeighborsClassifier. According to the results of repeated random trials, we chose the CatBoostClassifier, which performed best. The area under the ROC curve (AUC), known as the c‐statistic, explained the probability of a people with HCM having a higher model predictive value. SHAP methods were used to provide insights into the importance of each variable model gene in HCM [30]. The SHAP values were calculated to compute the contribution of each parameter to the performance of the prediction model.

### 2.9 Western Blotting

Total protein from human myocardial tissues was extracted using RIPA buffer (Beyotime, P0013B), and the protein concentration was measured with a bicinchoninic acid (BCA) assay kit (Thermo, 231,227). Proteins (15 μg per sample) were separated by 10% Tris‐glycine gel electrophoresis and subsequently transferred onto a PVDF membrane. The membranes were blocked with 5% skim milk in TBST and incubated overnight at 4°C with the following primary antibodies: anti‐ITPR2 (1:1000, ER1803‐65‐10ul, HUABIO), anti‐PDGFB (1:1000, HA722003, HUABIO), anti‐GAPDH (1:5000, 60,004‐1‐Ig, Proteintech), anti‐CDH5 (1:2000, 83,766‐6‐RR, Proteintech) and anti‐TJP1 (1:5000, 21,773‐1‐AP, Proteintech). After three washes with TBST, the membranes were incubated with secondary antibodies (1:2000, SA00001‐1, SA00001‐2, Proteintech) for 1 h at room temperature and developed in a dark room. Quantitative analysis of western blot data from three independent experiments was performed using ImageJ software (National Institutes of Health, Bethesda, MD, USA).

### 2.10 RNA Extraction and Real‐Time PCR

Total RNA was extracted from cultured human peripheral blood mononuclear cells (PBMCs) using TRIzol reagent (Thermo Fisher Scientific, 15,596,026), following the manufacturer's protocol. The extracted RNA (1 μg) was reverse‐transcribed into complementary DNA (cDNA) using the Reverse Transcription Reagent kit (AG11706, AG) according to the provided instructions. Real‐time quantitative PCR (qPCR) was performed using a fluorescent qPCR instrument (Analytik Jena qTOWER3G, Germany) in conjunction with SYBR Green PCR Master Mix (AG11701, AG). Each sample was analysed with 3–6 biological replicates. Cycle threshold (CT) values were obtained using the Analytik Jena PCR System software, and data were analysed via the comparative CT method. GAPDH was used as the internal reference gene to normalise for variations in cDNA loading. Primer sequences used for qPCR are provided in the Data Supplement.

### 2.11 Immunofluorescence Staining

Immunofluorescence staining was conducted following previously established protocols [34]. In brief, tissue sections were fixed in 4% paraformaldehyde for 15 min and permeabilized with 0.1% Triton X‐100 in PBS for 30 min. After three washes with PBS, the sections were blocked with 10% BSA and incubated overnight at 4°C with primary antibodies against CDH5 (1:150, 83,766‐6‐RR, Proteintech) and ZO‐1 (1:150, 21,773‐1‐AP, Proteintech). Following incubation, the sections were washed and treated with fluorescent‐labelled secondary antibodies (1:500, ab150116, Abcam) for 1 h at room temperature in the dark. Nuclei were counterstained with DAPI. Fluorescence imaging was then performed on a Nikon A1R upright confocal microscope (Japan).

### 2.12 Statistics

The differential expression analysis of snRNA‐Seq data relied on the nonparametric Wilcoxon rank‐sum test. Marker genes were identified based on a minimum log‐fold change threshold of 0.25 and *p* values were computed using a Wilcoxon rank‐sum test. Student's *t*‐test was used for the comparison of two groups, and one‐way ANOVA, post hoc or two‐way ANOVA was used for the comparison of eight ECs subgroups in the manuscript. All results are expressed as means ± SD, and *p < 0.05* was considered statistically significant.

## 3 Results

### 3.1 Abnormal Endothelial Barrier Function in Left Ventricular of Patients With HCM

We conducted a reanalysis of two datasets of snRNA‐seq obtained from humans. The human dataset included samples from 19 health heart transplant donors and 25 patients diagnosed with HCM. A total of 35,369 nuclei of EC (HCM: 24053; health: 11,316) were sequenced. To define cell type‐specific markers for each cluster, we selected the top genes that showed the most representative expression compared to other cells. The ECs were ultimately divided into eight subpopulations, which were clustered into Art EC (SEMA3G, DLL4), Vein EC (PLVAP, IL1R1), Lymp EC (PROX1, TBX1) and capillary endothelium (Figure 1A). Based on the function of the capillaries, the capillary endothelium was subdivided into Cap Art EC (PIK3R3, CXCL12), Cap1 EC (KDR, PDGFD), Cap vein EC (IGFBP5, CALCRL), Cap2 junction (CA4, RGCC), Cap3 angiogenesis (FLT1, ENG) (Figure 1B). We used GJA5, CXCL12, SEMA6D, IGFBP5, CA4, PLAT, UNC5B and PROX1 to characterise the distribution of subpopulations by t‐SNE plot. (Figure 1C).

To further explore the differences between health and HCM groups, we conducted an enrichment analysis of the DEGs between the two groups. The gene ontology (GO) enrichment analysis revealed the involvement of cellular junctional pathways, including TJs, GJs, and adherence junctions in this process (Figure 1D,E, Figure S1C,D). Additionally, several biological processes were found to be enriched, such as cellular senescence, ECM‐receptor interaction, intracellular signal transduction, and cell communication (Figure S2). Then, we conducted gene set enrichment analysis (GSEA) pathway enrichment analysis of each subgroup using the clusterProfiler R package (Figure 1F, Figure S3). The GSEA results of the subgroups also showed enrichment of gap junction, tight junction and adherens junction.

### 3.2 Endothelial Junction Function Being Disrupted in the Early Stage of HCM via Pseudotime Analysis

**FIGURE 2:**
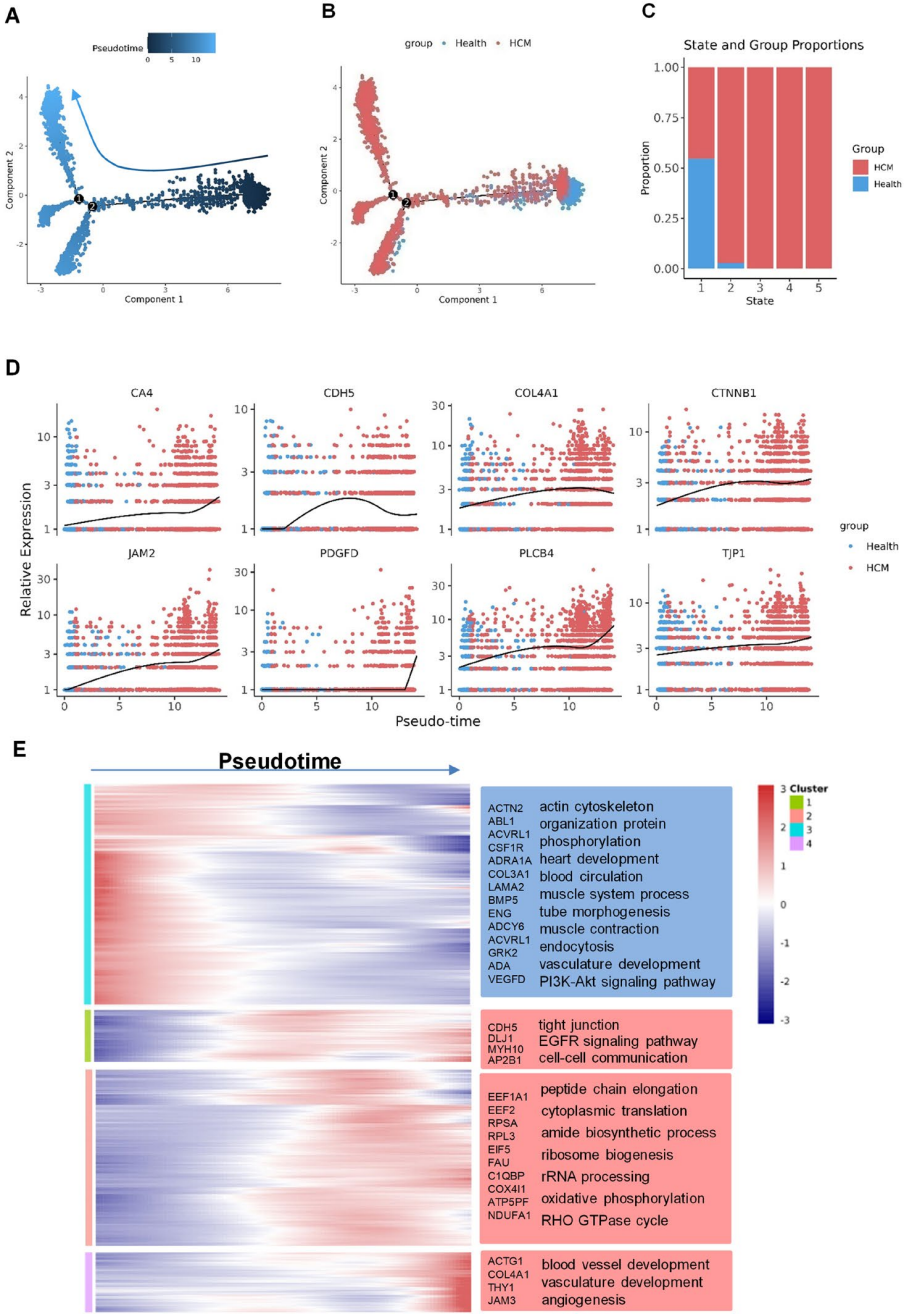
Pseudotime analysis Cap2 junction ECs in disease development trajectory and state process (A) Pseudotime trajectory prediction, the darker end is the starting point of the predicted timing development, and the lighter one is the end of the unpredicted development. (B) Comparison of proposed time trajectories between groups, red colour for the HCM group, blue for the health group. (C) Proportional stacked bar plot of inter‐group comparisons in pseudotime states. The *x*‐axis represents the pseudotime states, which are divided into five states. The *y*‐axis represents the proportion, displaying the ratio of the health group to the HCM group for each state. (D) Genes that change as a function of pseudotime. The *x*‐axis depicted the pseudotime progression, with group membership indicated by colour. The health group was denoted by blue, HCM group was represented by red. The *y*‐axis showcased the relative gene expression levels. (E) Pseudotime‐based gene clustering pathway analysis. The four clusters were indicated by the four colours on the left, the heatmap exhibited the relative expression levels of genes. The *x*‐axis of the heatmap depicted the progression of pseudotime, with red colour at the right end signifying upregulated genes towards the end of the pseudotime trajectory. The representative genes enriched in each cluster's pathways were listed.

In the above analysis presented, we observed an elevated proportion of Cap2 junction EC subpopulations in the HCM group (Figure S1A). In addition, the intergroup enrichment analysis enriched the cell junction pathways. Therefore, we hypothesized that cell junction pathways were involved in the development of the disease. We next explored the cell junction states in Cap2 junction ECs by inferring the state trajectories using Monocle2 (Figure 2A). We next integrated the proposed chronology of the health and HCM groups. The analysis revealed that the health group was undergoing development during the early stage of the pseudotime phase, while the HCM group exhibited development during the late stage of the pseudotime phase (Figure 2B). The proposed pseudotime analysis further divided the Cap2 junction subpopulation into five states, the 3–5 states were predominantly occupied by the HCM group (Figure 2C). These findings suggested that capillaries, particularly the Cap2 ECs, underwent unique stages in cardiac development compared to health groups.

We identified marker genes and cell junction‐related genes of the Cap2 junction ECs changed as a function of pseudotime (Figure 2D, Figure S4). Notably, CDH5, CTNNB1, JAM2 and TJP1 were identified as tight junction markers [35], while PDGFD and PLCB4 were identified as gap junction markers. The expression of genes associated with cell junctions, including TJs and GJs, increased as the pseudotime progressed. This suggested that cellular junction pathways and this biological process played a crucial role in the stages of disease progression.

To further explored the biological significance of different stages during the pseudo‐temporal sequence, we performed clustering enrichment analysis based on the pseudo‐temporal expression patterns of genes. Cluster 1 genes showed elevated expression at the initial stage of pseudo‐temporal development and decreased at the advanced stage of disease progression. In contrast, Cluster 2, 3 and 4 genes exhibited higher expression levels during the later stage of disease evolution and were downregulated at the initial stage (Figure 2E). The Cluster 2 genes were enriched in pathways related to TJs, the EGF/EGFR signalling pathway and cell–cell communication. Cluster 3, which included a larger fraction of genes, was predominantly enriched in pathways related to amino acid and nucleic acid production metabolism, such as peptide chain elongation, cytoplasmic translation, and amide biosynthetic processes. Additionally, we employed another pseudotime analysis method, Slingshot, which is based on a minimum spanning tree (MST) graph algorithm combined with simultaneous principal curve fitting. Slingshot calculates pseudotime values by determining the projection distance of each cell onto the curve [33]. Through Slingshot analysis of the entire endothelial cell population, we observed that cap2 junction ECs were positioned at an intermediate pseudotime state between arterial and venous ECs. This result aligns well with the pseudotime analysis obtained from the Monocle method (Figure S10). In conclusion, our findings indicate that TJs, angiogenesis, cell–cell communication, and vasculature development are upregulated during the early stages of disease progression in the Cap2 junction ECs. Otherwise, we also observed the same phenomenon in the entire endothelial cell population (Figure S5).

### 3.3 Validation of Endothelial Barrier Function During the HCM Progress via Fate Mapping Technique

We used mouse snRNA‐seq data to validate the above findings We obtained tdTomato mouse lines from the GSE166403 dataset. The transgenic mouse lines expressing CreER;tdTomato (Cdh5‐CreER) were treated with tamoxifen and then underwent either Sham or TAC surgery. Ultimately, the mice were categorised into three groups: Sham group (*n* = 3), TAC 2w group (*n* = 3), and TAC 4w group (*n* = 4).

**FIGURE 3:**
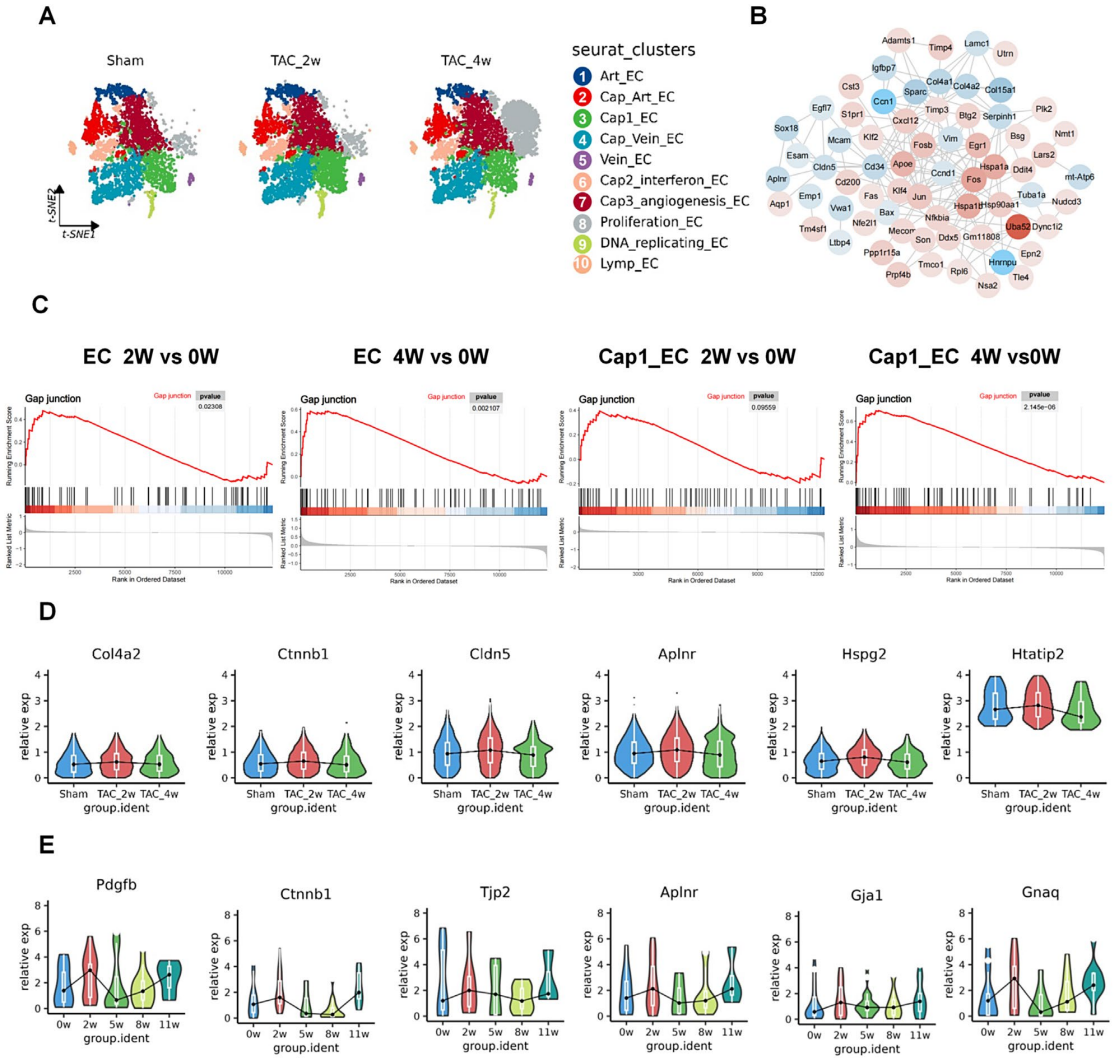
Fate tracing reveals cell junction changes during the progression of HCM (A) tSNE plot of Cdh5CreeER; tdtomato TAC mice in 0, 2 and 4 weeks after sham or TAC surgery. Ten colours coded for 10 subcluster types. (B) Gene network topological map. The genes represent the intersection between the TAC 2 W versus Sham groups and the TAC 4 W versus Sham groups. The colour changes indicate the magnitude of log fold change (logFC). The red colour represents logFC > 0, with darker shades indicating larger values. The blue colour represents logFC < 0. (C) GSEA curves demonstrate pathway enrichment scores for gap junction pathways at 2 and 4 W. The blue portion indicates the gene set is predominantly expressed at lower levels across the entire sample, while the red portion indicates higher expression levels of the gene set within the sample. (D) Violin and box plots showing the relative expression levels of cell junction genes at 0, 2 and 4 weeks. (E) Violin and box plots showing the relative expression levels of cell junction genes at 0, 2, 5 8 and 11 weeks in GSE120064 data.

Based on the characteristics of lineage tracing, which allows specific targeting of ECs, we naturally divided the ECs into 10 subpopulations: (Figure 3A). In both TAC 2w and TAC 4w groups, we found 68 DEGs in the ECs compared to the Sham group (Figure 3B), the intensity of colours represents the fold change (FC) values.

To investigate the changes in cell junction pathways over the 4 weeks, we performed a GSEA analysis of DEGs between the 2 W versus 0 W and 4 W versus 0 W groups for ECs and Cap1 ECs. In ECs the *p*‐adjust value for GJs was 0.02308 at 2 weeks and 0.002107 at 4 weeks. Similarly, in Cap1 ECs, the *p*‐value for GJs was 0.09559 at 2 weeks and 2.145e‐06 at 4 weeks (Figure 3C). The results indicated activation of the gap junction pathway during disease progression at 4 weeks. This also supported the hypothesis that 2 weeks may represent an early predictive window for the disease. To quantify and validate the hypothesis that 2 weeks can serve as an early predictor for the disease, we compared the expression levels of genes involved in cell junction pathways (Figure 3D). Genes such as Col4a2, Ctnnb1, Cldn5, Aplnr, Hspg2 and Htatip2 showed consistent trends: an increase from 0 W to 2 W followed by a decrease from 2 to 4 W. We also utilised the GSE120064 dataset, which includes representative time points (0, 2, 5, 8 and 11 weeks) of mice after TAC‐induced pressure overload. Genes related to cell junctions exhibited an abrupt increase during the early stage (0–2 weeks), followed by a subsequent decline over time. However, during the late stage (8–11 weeks), they showed a renewed rise (Figure 3E). These findings, consistent with the previous dataset, underscored the potential of cell junction genes as a screening approach for early detection of diseases.

### 3.4 Cellular Communication and Transcription Factors Contribute to Endothelial Barrier Function

**FIGURE 4:**
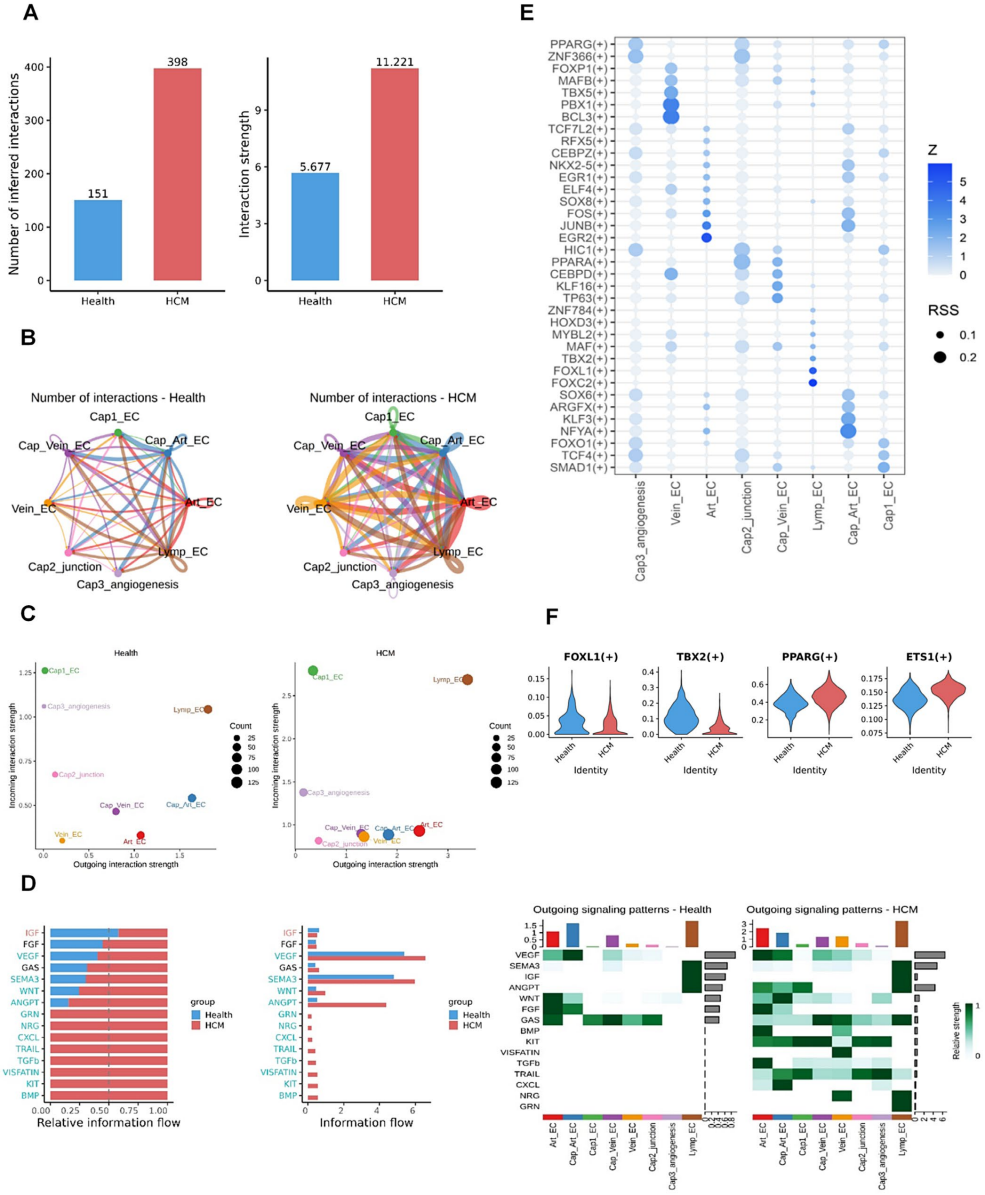
Endothelial cell communication and transcription factor analysis (A) Barplot of signal interactions, the vertical coordinates were the total number of signal interactions and the sum of intensities. (B) Differences in the number and strength of interactions between cell clusters. Red edges indicated increased signal connections in the second dataset, while blue edges symbolised decreased interactions. (C) Compared to the major sources and targets in 2D space, the *x*‐axis represented the strength of outgoing interactions, while the *y*‐axis represented the strength of incoming interactions. (D) Comparison of inter‐group and intra‐subgroup signal flow in signalling pathways. Inter‐Group Comparison: Distinctions between normal and disease groups in signalling pathways, displayed the proportion of each pathway and ranking of significant pathways. Intra‐subgroup comparison: Compared outgoing signals associated with each subgroup. (E) Transcription factor activity analysis among subgroups. *Z*‐values represent a statistical measure of the deviation of transcription factor expression within single cells. RSS (residual sum of squares) indicates the variability of transcription factors within single cells. (F) Transcription factors with significant differences between groups.

**FIGURE 5:**
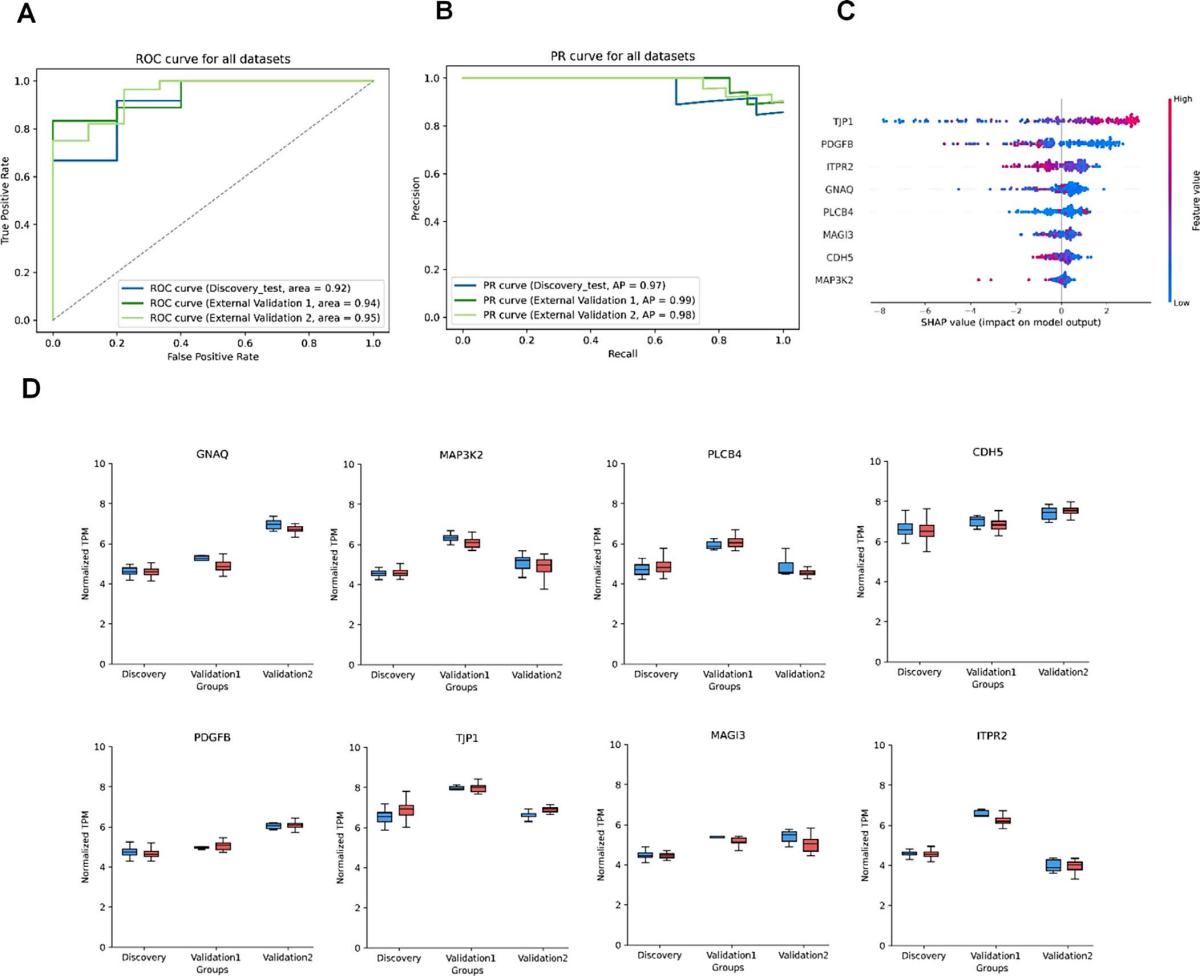
Early risk prediction model for hypertrophic cardiomyopathy (A) Performance evaluation of classifier by receiver operator characteristic curve (ROC) for early risk prediction of hypertrophic cardiomyopathy in three Datasets. (B) Evaluation of precision‐recall curve for the performance assessment of the model. (C) Assessment of cell junction gene rankings in the SHAP model importance. The greater the contribution to the model, the higher the score it received. (D) The mRNA expression levels of 8 genes were used for the prediction of HCM. These genes were represented by normalised transcripts per million (TPM) values across the discovery cohort (39 normotensive individuals and 106 HCM patients), validation 1 cohort (5 normotensive individuals and 18 HCM patients), and validation 2 cohort (8 normotensive individuals and 28 HCM patients).

Cellular communication is the fundamental nature of cell junctions [36]. Through the aforementioned pseudotime and gene enrichment analyses, we have identified the significant role of cell junction pathways in HCM disease progression. To investigate the underlying mechanisms, particularly in specific subgroups of EC cells with distinct and extensive communication pathways, we performed cell communication analysis on the human dataset. we observed that both the total number and strength of cell interactions were higher in the HCM group compared to the health group (Figure 4A). We constructed a cell communication network among the EC subgroups, revealing increased cell communication in HCM groups compared to the health group (Figure 4B, Figure S6A,B). The differences in communication between subgroups were mainly observed in Cap Art EC, Art EC and Lymp EC subgroups in the HCM group, they had significantly more cellular communication than the health group. When comparing the major sources of communication in the 2D space, we found that the Cap3 angiogenesis ECs and Cap2 junction ECs showed the most prominently decrease in incoming interaction strength (Figure 5C).

Next, we investigated the specific communication signals that contributed to these changes. In the comparison between HCM and health groups, several pathways, such as GNR, CXCL, TRAIL, TGFβ, VISFATIN and KIT, were found in the HCM group but not in the health group. Among the interactions between different signalling pathways, VEGF, SEMA3 and ANGPT showed the highest levels of interaction (Figure 5D, Figure S6C,D). In the HCM group, ANGPT and VEGF signals were more abundant in the Art EC, Cap Art EC and Cap1 EC subgroups (Figure S6E,F). Otherwise, in the health group, the FGF pathway exhibited more communication in the Art EC and Cap Art EC subgroups. Supporting Information provided details on the interactions between ECs and other cell types. (Figure S7).

In the context of HCM disease progression, with such significant changes in cell communication, the involvement of transcription factors is undoubtedly crucial. We conducted a transcription factor analysis using the SCENIC method for various subgroups of ECs (Figure 4E, Figure S8). Each subgroup demonstrated its own set of highly expressed and specific transcription factors. For example, the vein ECs exhibited high expression of transcription factors such as BCL3 and PBX1, and the Art ECs were predominantly regulated by EGR2 and JUNB. Furthermore, The Cap2 junction ECs, showed significant regulation by transcription factors such as HIC1, CEBPD and PPARA. Among the transcription factors, some were downregulated in the disease group, such as TBX2 and FOXL1, while others were upregulated, such as PPARG and EST1 (Figure 5F).

### 3.5 Early Risk Prediction of HCM With a Panel of 8 Cell Junction Genes

In both human and mouse datasets, we identified subtle roles of cell junction pathways and genes in HCM. Particularly, in the mouse dataset, we discovered the phenomenon that 2 W cell junctions could serve as an early predictive factor. To further validate the potential of HCM‐related genes as indicators for early disease screening, we constructed a gene‐based diagnostic prediction model using three HCM transcriptome datasets, including one training set and two external validation sets. The training of the model utilised mRNA data from the discovery cohort, consisting of 39 health donors and 106 patients diagnosed with HCM (GSE36961). After comparing 12 machine‐learning classification models, we employed the CatBoost classifier for our analysis (Table S1). In repeated random validations, the final model exhibited strong performance. The area under the receiver operating characteristic curve (AUROC) approached perfection at 0.92, indicating excellent discrimination ability. The area under the precision‐recall curve (AUPRC) achieved a perfect score of 0.97, demonstrating high precision. The accuracy was 0.81 [0.63–0.98], Subsequently, we performed validation of our model on two separate cohorts: External Validation 1 (GSE160997), which included 5 healthy donors and 18 HCM patients, and External Validation 2 (GSE130036), which consisted of 8 healthy donors and 28 HCM patients. We then tested this model on validations, each validation set underwent 200 iterations of random sampling to ensure robustness and reliability. The model demonstrated impressive performance metrics on both validation sets (Figure 5A,B, Table S2), achieving an AUROC of 0.94 and 0.95, AUPRC of 0.99 and 0.98, and accuracy of 82% [67%–97%] and 83% [70%–95%] for validation 1 and 2, respectively, within a 95% confidence interval. Later, we incorporated the 8 cell junction genes into the SHAP model and obtained their importance rankings. Among the highly important genes in the overall predictive model were TJP1, PDGFB, ITPR2, GNAQ, PLCB4 and others (Figure 5C).

We used the panel genes to show the effectiveness of the model. It was observed that the genes showing consistent trends between the validation and training sets were also validated in the training set (Figure 5D). Even when expanding the panel to include 19 cell junction genes, our model still achieved satisfactory predictive performance for disease classification (Figure S9). Certainly, these findings further solidify the diagnostic potential of these genes in HCM, offering additional evidence to support their role as prospective diagnostic biomarkers and targets for investigating the disease mechanism. These discoveries are expected to offer new directions and strategies for the early diagnosis and treatment of HCM.

### 3.6 Aberrant Cell Junction Genes in Hypertrophic Cardiomyopathy

**FIGURE 6:**
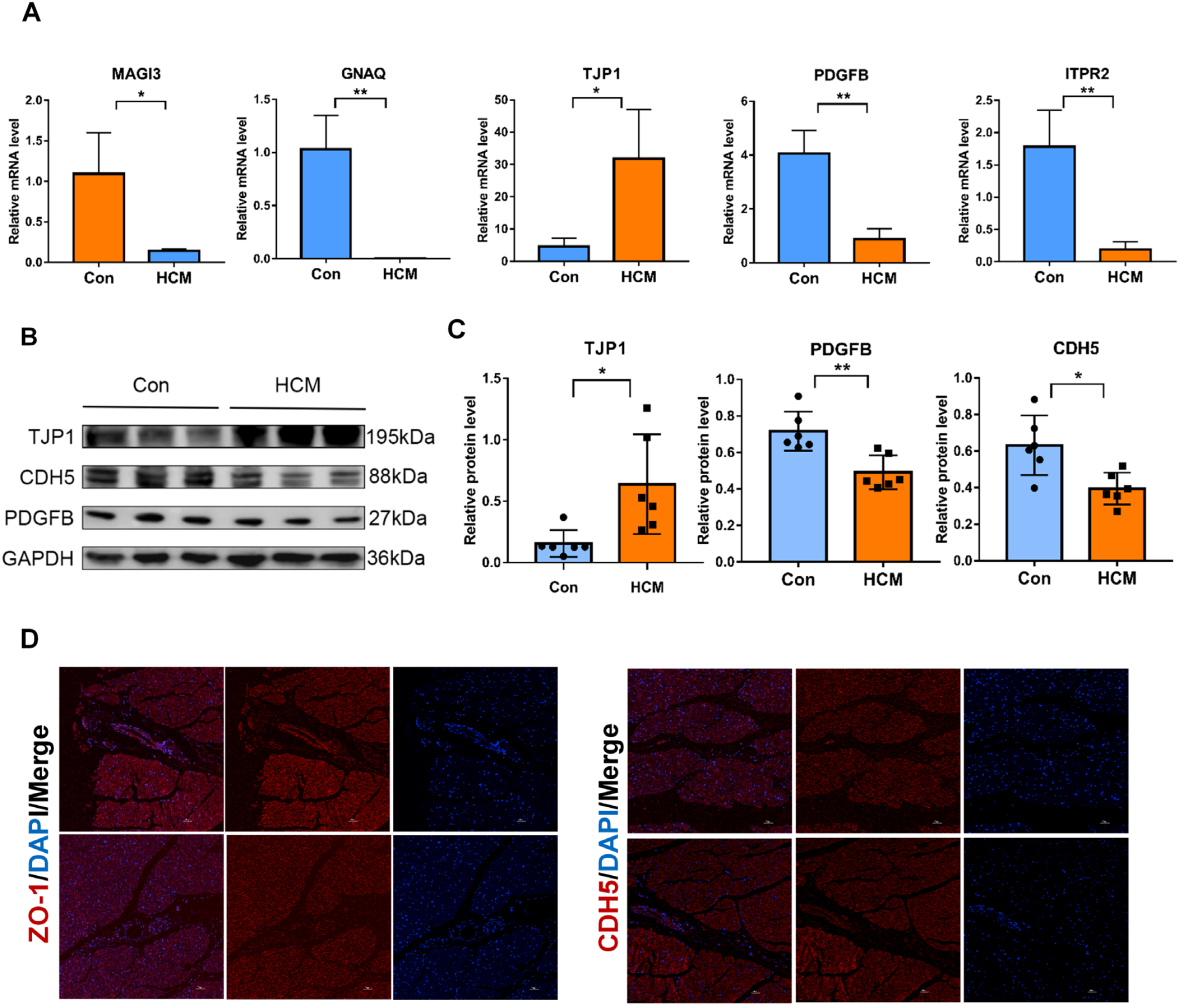
Abnormal expression of the cell junctions in hypertrophic cardiomyopathy (A) Transcriptional levels of cell junction genes in human PBMCs were detected using real‐time quantitative polymerase chain reaction (qPCR). *p*‐values were calculated by unpaired Student's *t*‐test. *N* = 3; *p* < 0.05 (*), *p* < 0.01 (**). (B, C) Western blot analysis showing expression levels of cell junction‐associated markers in human myocardial tissues. *N* = 6; *p* < 0.05 (*), *p* < 0.01 (**). (D) Immunostaining illustrating the expression of ZO‐1 and CDH5. Nuclei were counterstained with DAPI. Scale bar, 50 μm.

To validate the accuracy of the aforementioned model regarding the role of cell junction genes in disease diagnosis, we conducted biological verification. First, we collected blood samples from HCM patients and healthy individuals. After extracting PBMCs, we compared the mRNA expression levels of TJP1, PDGFB, ITPR2, GNAQ and MAGI3 between patients and healthy controls. The results were consistent with our bioinformatics findings, showing that TJP1 was elevated in HCM, while PDGFB, ITPR2, GNAQ and MAGI3 were expressed at lower levels compared to healthy individuals. We further confirmed these findings at the protein level (Figure 6A). In myocardial tissues from HCM patients and healthy individuals, the protein ZO‐1 (zonula occludens‐1), encoded by the cell junction gene TJP1, was significantly upregulated in HCM, while CDH5 and PDGFB were both downregulated (Figure 6B,C). Previous studies have shown that in dilated cardiomyopathy (DCM), the remodelling of ZO‐1 is regulated, which accelerates disease progression [37]. Immunofluorescence staining of pathological tissue samples further validated the expression levels of TJP1 and CDH5 (Figure 6D). In conclusion, combining experimental validation, model construction, and multi‐omics analysis, we found that cell junctions, especially endothelial cell junctions, play a crucial role in the progression of HCM and could potentially serve as therapeutic targets for HCM.

## 4 Discussion

Currently, the changes of ECs in the pathogenesis of HCM are not well investigated. In our analyses, we identified cell junction changes at snRNA‐seq resolution in human data as well as mice data. First, we presented a comprehensive cellular composition of HCM ECs. then we identified abnormal alterations in cell junction pathways in ECs and provided novel insight into how the cell junction genes expression landscape was altered in ECs. Next, we performed cellular communication analysis and transcription factor analysis to uncover potential intrinsic correlations. We ultimately proposed an HCM diagnostic model for early detection.

ECs have long been suggested to represent a heterogeneous population [38]. Studies have suggested that microvascular dysfunction in HCM models may arise as a result of impaired myocardial capillary growth during the early postnatal period and can precede the development of myocardial hypertrophy [39]. HCM patients showed an increased mobilisation of EPCs compared with healthy individuals that correlated with diastolic dysfunction. Endothelial progenitor cells (EPCs), originating from the bone marrow, are mobilised into the bloodstream where they migrate and differentiate into mature ECs, playing a crucial role in both physiological and pathological neovascularization after birth [40]. It is worthwhile for us to further investigate the function of ECs, particularly in the process of disease development. In the comparison between HCM and health, there was an upregulation in the proportion of Art EC, Cap1 EC and Cap2 junction, while the proportion of Cap3 angiogenesis showed the most significant downregulation (Figure S1A,B). Previous studies have reported that regulation of angiogenesis is an important process in cardiac remodelling and increased angiogenesis prevents functional decline in heart failure [41]. Increased angiogenesis occurs early during cardiac remodelling while later transitions into capillary dysfunction and capillary loss [42]. GO functional enrichment analysis of DEGs between HCM and health revealed significant enrichment in cellular junction pathways, such as gap junction, tight junction and adherens junction. A separate study demonstrated that the deficiency of a disintegrin and metalloproteinase‐17 (ADAM17) in ECs preserves the expression of AJs and TJs, thereby protecting the integrity of the intimal barrier and suppressing the formation of thoracic aortic aneurysm [43]. Also, the breakdown of endothelial cell‐to‐cell junctions is the main reason for blood‐retinal barrier dysfunction [44]. TJs are located at the apicolateral region, sealing the intercellular space, while AJs are located basally, following or intermingling with TJs, and providing strong mechanical connections between neighbouring cells. TJs are involved in the regulation of cellular functions (e.g., proliferation, differentiation and apoptosis) due to their signal transduction abilities [45, 46]. While GJs facilitate intercellular communication [47]. Consequently, they serve as an essential barrier in blood vessels, contributing to the maintenance of vascular homeostasis [48]. The disruption of intercellular junctions linked to myofibre disarray in HCM is likely to play a critical role in the disease's pathophysiological manifestations. This remodelling of GJs may create an arrhythmogenic substrate, contributing to the development and persistence of cardiac arrhythmias commonly seen in HCM [49]. We doubted that TJs, AJs and GJs underwent significant changes during the progression of HCM.

In contrast to DCM, HCM rarely progresses to systolic dysfunction or reaches end‐stage disease [50, 51]. In contrast to DCM, hypertrophic cardiomyopathy (HCM) rarely progresses to systolic dysfunction or reaches end‐stage disease. In the pseudotime trajectory of gene expression, the marker genes of the cap2 junction cluster and other cell junction‐related genes showed an increase in expression during the later stages of the time course. For instance, the COL4A1 study demonstrated similar findings previously reported in mice with Col4a1 mutation including collagen disarray and reduction of electron density in the basement membrane of capillary ECs and muscle fibres. The study adds another disease spectrum of COL4A1 mutation which include HCM [52]. Research also reported that TJP1, IGFBP5 and CREB5 may form an important network involved in the pathology of HCM [53]. Consistently, the pseudotime pathway heatmap indicated frequent alterations in cell junctions and cell communication during the early stages of disease progression. Pathological cardiac hypertrophy is a common predecessor to heart failure [23]. We found that at 4 weeks (late stage), the cell junction pathways showed greater statistical significance compared to 2 weeks (early stage). Research manifested that the captured 2‐week time point in the study likely results from the early acute phase of compensatory mechanisms, but is not maintained until the 4‐week time point [24]. We validated this phenomenon using genes associated with cell junction pathways, such as Cldn5, Col4a2, Ctnnb1 and others. Although the TAC mouse model does not fully replicate the pathophysiological characteristics of HCM or the hereditary gene mutations associated with the condition, it does share similarities. More importantly, studies have demonstrated a causal relationship between TAC mice and human HCM populations. Researchers have confirmed this through both in vivo and in vitro experiments, providing further evidence of this connection [54]. Direct comparisons have shown that cardiac hypertrophy experimentally induced by TAC, leading to chronic pressure overload, is associated with reduced capillary density [55]. This mirrors key aspects of HCM pathophysiology. HCM is characterised by collagen deposition, which is a hallmark of the disease. Studies have shown that pressure‐overloaded mice develop myocardial fibrosis, with significant collagen accumulation observed during disease progression [56]. In brief, this model closely mimics the disease progression seen in HCM.

Cell communication is a fundamental biological process underlying the enrichment of cell junction pathways [31]. We not only observed higher cell interaction intensity and frequency in the HCM group compared to the health group but also identified a significant decrease in the incoming signal strength of the cap2 junction ECs, along with the emergence of multiple unique signalling pathways specific to the HCM group, such as VEGF signalling, SEMA3 signalling and ANGPT signalling. Research found that the VEGF signalling pathway prevented apoptosis and preserved contractile function in hypertrophied infant heart [57]. In the transcription factor analysis, we observed a high activity of PPARA (+) in the cap2 junction subgroup. Notably, multiple studies have shown suppressed PPARA expressions in DCM and HCM hearts carrying different genetic variants [58, 59]. However, the cardioprotective effects of ligand‐activated PPARA have been reported, including the restored balance between fatty acid uptake and FAO, increased insulin sensitivity, reduced ROS production and attenuated fibrosis formation [60, 61]. Based on the above findings, PPARA may be a novel target involved in the regulation of the cell junction pathway in HCM. This study demonstrates that there is robust intercellular communication, particularly between arterial ECs and lymphatic ECs, which interact with other endothelial cell subpopulations through intercellular pathways such as VEGF and FGF. Additionally, interactions both intracellularly and extracellularly are equally important. for instance, extracellular vesicles (EVs) are small membranous vesicles that contain a diverse array of RNA species, each with specialised functions and clinical implications [62, 63, 64]. EVs are known to encapsulate various RNA species with specialised roles and clinical significance. Furthermore, EVs hold substantial diagnostic potential in disease contexts. Research has indicated that plasma extracellular vesicle long RNAs may serve as biomarkers for the early detection of colorectal cancer, small cell lung cancer, and other diseases [65, 66, 67]. Therefore, further investigation is warranted to determine the diagnostic value of EVs in HCM.

To investigate their potential for early detection of HCM, we utilised clinical mRNA data and developed a machine‐learning classification model based on a panel of cell junction genes. Among the genes included in our model, the RAF1 gene was found that a direct link between the gene variant and the abnormal sarcomere structure resulting in a cardiac dysfunction that remarkably recapitulates the HCM [68]. AKT3 plays a crucial role in the mechanism by which CM‐specific mir15a/mir16‐1 knockout promotes cardiac hypertrophy and dysfunction after TAC [30, 54]. The application of this diagnostic model provides broad prospects for early risk screening of HCM. By utilising cell junction genes as predictive markers, the model can accurately distinguish between healthy individuals and those with HCM. The TJP1 gene encodes the ZO‐1 protein, which has been implicated in the dysfunction of TJs in various diseases, such as aortic dissection and non‐small cell lung cancer (NSCLC) [16, 69]. Interestingly, ZO‐1 has been found to enhance the transcriptional regulation of caspase‐3, thereby promoting apoptosis. This represents a previously unrecognised anti‐apoptotic mechanism that aids in maintaining mucosal homeostasis under inflammatory conditions [70]. Another gene in the panel, ITPR2, has been identified as a driver of cellular senescence and aging through its role in calcium channel activity, facilitating contacts between mitochondria and the endoplasmic reticulum. Furthermore, ITPR2 can mitigate cell necrosis induced by aflatoxin [71]. Regarding the GNAQ gene, evidence suggests that GNAQ mutations can lead to the excessive activation of intracellular calcium signalling in ECs, triggered by both constitutive and G protein‐coupled receptor ligand‐induced mechanisms. These findings may be relevant to the disease progression of HCM [72]. The diagnostic panel we have identified could be developed into a diagnostic reagent kit in the future, particularly targeting high‐risk populations, such as individuals with a family history of HCM. In clinical practice, blood samples will be collected from patients to assess the RNA levels of selected cell junction‐related genes, with the results inputted into predictive models. This approach may provide valuable insights for both high‐risk individuals and patients in the disease progression stage. In future research, we aim to conduct more in‐depth studies on cell junction genes, exploring the pathogenicity of individual genes and designing drug targets, thereby contributing to new diagnostic and therapeutic strategies for the precise treatment of HCM.

In summary, we utilised snRNA‐Seq data to analyse the endothelial cell atlas of HCM and discovered the subtle role of cell junctions in the disease. Subsequently, we confirmed the activation and upregulation of cell junctions during the early to late stages of disease progression using pseudotime analysis and lineage‐tracing mouse models. By analysing cell communication and transcription factors, we explored the potential mechanisms underlying their involvement. Finally, we established a predictive model for the disease using cell junction genes and machine learning methods. The diagnostic model serves as a powerful tool for researchers and clinicians to assess individual HCM risk. In conclusion, the application of this diagnostic model has significant clinical implications and potential for clinical utility, offering promising improvements in the early diagnosis of HCM.

## Author Contributions

**Dingchen Wang:** formal analysis (equal), visualization (equal), writing – original draft (lead). **Xiaoran Huang:** methodology (supporting), supervision (supporting). **Yaowen Liang:** conceptualization (equal), data curation (equal), methodology (equal). **Xiran Wang:** methodology (equal), project administration (equal). **Yuge Chen:** methodology (equal), writing – original draft (supporting). **Yunfei Gao:** writing – review and editing (equal). **Miao Lin:** formal analysis (equal), funding acquisition (equal), visualization (equal). **Huiying Liang:** funding acquisition (equal), writing – review and editing (equal). **Xin Li:** funding acquisition (lead), investigation (equal). **Ruobing Wang:** data curation (equal), methodology (equal). **Huiming Guo:** data curation (equal), methodology (equal).

## Conflicts of Interest

The authors declare no conflicts of interest.

## Supporting information


Figure S1.

**Table S1.** Twelve machine‐learning classification models for hypertrophic cardiomyopathy.

**Table S2.** Validation and performance assessment of the machine‐learning classification model.

## Data Availability

The snRNA sequencing data reported in this study are available in the National Center of Biotechnology Information Gene Expression Omnibus database: GSE120064 and GSE166403 and in the Broad Institute's Single Cell Portal project ID SCP1303. Transcriptomic datasets reported in this study are available in GSE36961, GSE160997 and GSE130036.
