# Supplementary material for: Single‐Cell Transcriptomic Reveals the Involvement of Cell–Cell Junctions in the Early Development of Hypertrophic Cardiomyopathy
Source: J Cell Mol Med. 2025 Feb 3;29(3):e70366. doi: 10.1111/jcmm.70366 (PMC11790354; doi:10.1111/jcmm.70366)
Supplement: Supplementary file 1 — Figure S1. [file JCMM-29-e70366-s002.docx]

Supplemental Materials

**Single-cell transcriptomic reveals the involvement of cell-cell junctions in the early development of hypertrophic cardiomyopathy**

Dingchen Wang^1,2†^, Miao Lin^3,4,5†^, Ruobing Wang^3†^, Xiaoran Huang^2^, Yaowen Liang^2,9^, Xiran Wang^3,5^,Yuge Chen^6^, Yunfei Gao^7,8^, Huiming Guo^3*^, Huiying Liang^3,4,5*^, Xin Li^1,2^^*^

**Affiliations**

1. School of Medicine, South China University of Technology; Guangzhou, Guangdong Province, 510006, China.

2. Department of Emergency Medicine, Guangdong Provincial People's Hospital (Guangdong Academy of Medical Sciences), Southern Medical University; Guangzhou, Guangdong Province, 510080, China.

3. Guangdong Cardiovascular Institute, Guangdong Provincial People's Hospital, Guangdong Academy of Medical Sciences; Guangzhou, Guangdong Province, 510080, China.

4. Medical Big Data Center, Guangdong Provincial People's Hospital (Guangdong Academy of Medical Sciences), Southern Medical University; Guangzhou, Guangdong Province, 510080, China.

5. Guangdong Provincial Key Laboratory of Artificial Intelligence in Medical Image Analysis and Application; Guangzhou, Guangdong Province, 510080, China.

6. Department of Obstetrics and Gynecology, The First Affiliated Hospital of Anhui Medical University, Hefei 230022, China.

7. Zhuhai Precision Medical Center, Zhuhai People's Hospital (Zhuhai Hospital Affiliated with Jinan University), Jinan University; Zhuhai, Guangdong Province 519000, China.

8. The Biomedical Translational Research Institute, Jinan University Faculty of Medical Science, Jinan University; Guangzhou, Guangdong Province 510632, China.

9. Shantou University Medical College; Shantou, Guangdong Province, 515041, China.

Dingchen Wang, Miao Lin, Ruobing Wang contributed equally to this study.

Correspondence:

Xin Li, MD, Department of Emergency Medicine, Guangdong Provincial People's Hospital (Guangdong Academy of Medical Sciences), Southern Medical University; Guangzhou, Guangdong Province, 510080, China.

Email: xlidoct@qq.com

Huiying Liang, MD, Medical Big Data Center, Guangdong Provincial People's Hospital (Guangdong Academy of Medical Sciences), Southern Medical University; Guangzhou, Guangdong Province, 510080, China.

Email: lianghuiying@hotmail.com

Huiming Guo, MD, Guangdong Cardiovascular Institute, Guangdong Provincial People's Hospital, Guangdong Academy of Medical Sciences; Guangzhou, Guangdong Province, 510080, China.

Email: guohuiming@gdph.org.cn.

Contents:

Figure S1–S9

**Figure. S1 Endothelial cell subpopulations and enrichment of differentially expressed genes** (A) The proportions of endothelial cell subpopulations in the disease and normal groups were visualized by color-coding, with each color representing a specific subpopulation. (B) The proportions of each endothelial cell subpopulation in the normal and disease groups. The blue color represents the normal group, while the red color represents the disease group. (C) DEGs enrichment analysis in groups. Differentially expressed genes were identified based on a logFC threshold of 0.25. Enrichment analysis was performed using the ClusterProfiler package, with the color intensity representing the significance of the p-values and the size of the scatter plot indicating the number of genes enriched in each pathway. (F). Enrichment analysis of differentially expressed genes in the eight subpopulations using GSEA. The size of the scatter points symbolized the significance level. The color of the points was filled based on the NES.

**Figure. S2 Pathway network topology in enrichment analysis between groups**

Different colors represent different groups. The connections represent the gene interactions between pathways.

**Figure. S3 Network Topology of Enrichment Analysis among Subgroups**

Different colors represent different subgroups. The connections represent the gene interactions between pathways.

**Figure. S4 Pseudotime analysis of cell junction genes in endothelial cell subtypes**

The x-axis represents the pseudotime progression, while the y-axis reflects the relative expression levels of genes. Different colors represent different subtypes.

**Figure. S5 Pseudotime analysis of cell connectivity in endothelial Cells**

(A) Pseudotime trajectory prediction, the darker end is the starting point of the predicted timing development, and the lighter one is the end of the unpredicted development. (B) Comparison of proposed time trajectories between groups, red color for HCM group, blue for health group. (C) Predicting cell states through pseudotime analysis. Pseudotime analysis divides cell states into seven stages, with each color representing a specific stage, and branching points indicating transitions between states. (D) Cluster analysis of cell states. The dendrogram illustrates the clustering relationship of cell states, revealing the dynamics of cell state transitions. The branches represent the hierarchical relationships between different categories. (E) Genes that change as a function of pseudotime. The x-axis depicted the pseudotime progression, with group membership indicated by color. Health group was denoted by blue, HCM group was represented by red. The y-axis showcased the relative gene expression levels.

**Figure. S6 Revealing signal interactions through cell communication in Cap2 Junction endothelial cells** (A-B) Differences in the number and strength of interactions between cell clusters. The intensity and number of lines represent the strength and quantity of interactions between cells. (C) The distance of signal propagation, with longer distances indicating more active signaling. (D) Chord diagram of the VEGF signaling pathway between groups. The thickness of chords reflects the strength of cell-cell communication in the VEGF signaling pathway. (E) Heatmap of incoming signals between subgroups between groups. The horizontal axis represents cell types, while the vertical axis represents signal categories. The bar chart displays the strength of the signal, and the color indicates the intensity of the signal within each subgroup. (F) Heatmap of overall signals between subgroups between groups.

**Figure. S7 Intercellular communication among different cell types in the Heart** (A) Differences in the number and strength of interactions between cardic cell clusters. Red edges indicated increased signal connections in the second dataset, while blue edges symbolized decreased interactions. (B) Intra-subgroup comparison: compared outgoing signals associated with each subgroup. (C) Comparison of inter-group signal flow in signaling pathways. Distinctions between normal and disease groups in signaling pathways, displayed the proportion of each pathway and ranking of significant pathways.

**Figure. S8 Comparison of transcription factor activity differences between normal and HCM groups**

Violin plot showing the expression of transcription factors in the HCM and health groups. Red represents the disease group, and blue represents the normal group.


**Figure. S9 Risk prediction early in HCM via a 19-gene cell junction panel**

(A) Performance evaluation of classifier by receiver operator characteristic curve (ROC) for early risk prediction of hypertrophic cardiomyopathy in three Datasets. (B) Evaluation of precision-recall curve for the performance assessment of the model. (C) Assessment of cell junction gene rankings in the SHAP model importance. The greater the contribution to the model, the higher the score it received. (D) The mRNA expression levels of 19 genes were used for the prediction of HCM. These genes were represented by normalized transcripts per million (TPM) values across the discovery cohort (39 normotensive individuals and 106 HCM patients), validation 1 cohort (5 normotensive individuals and 18 HCM patients), and validation 2 cohort (8 normotensive individuals and 28 HCM patients).


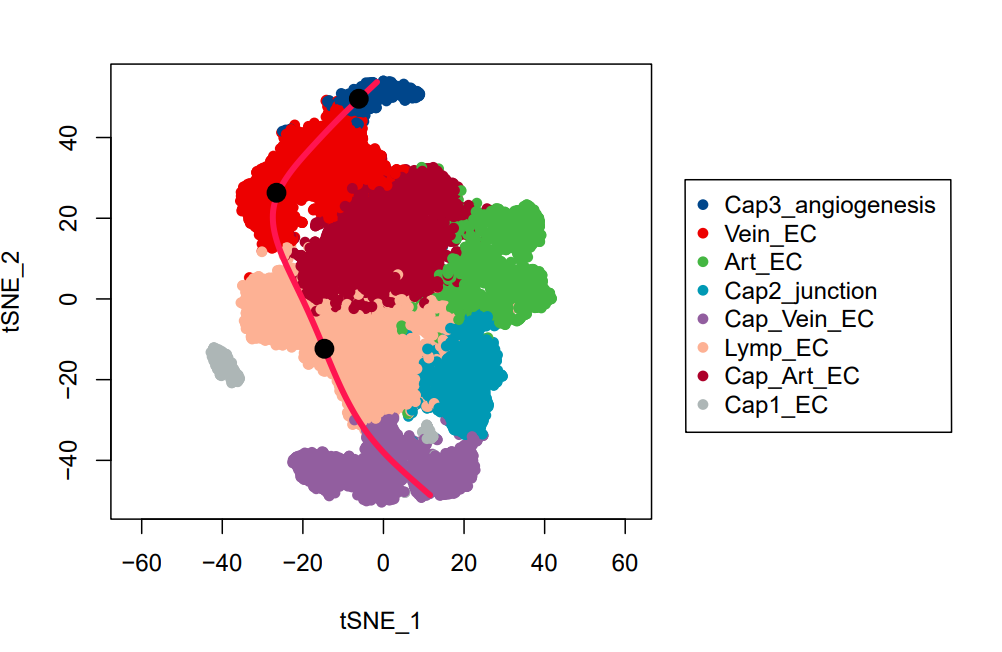


**Figure. S10 Slingshot pseudotime inference analysis**
